# Measurement of asthma control according to global initiative for asthma guidelines: a comparison with the asthma control questionnaire

**DOI:** 10.1186/1465-9921-13-50

**Published:** 2012-06-22

**Authors:** José María Olaguibel, Santiago Quirce, Berta Juliá, Cristina Fernández, Ana María Fortuna, Jesús Molina, Vicente Plaza

**Affiliations:** 1 Department of Allergy, Complejo Hospitalario de Navarra, Fundación Miguel Servet, Pamplona, Spain; 2 Department of Allergy, Hospital La Paz Health Research Institute (IdiPAZ), Madrid, Spain; 3 Medical Department, MSD, c/Josefa Valcárcel, 38, Madrid, Spain; 4 Epidemiology and Research Support Department, Hospital Clínico San Carlos, Madrid, Spain; 5 Department of Pulmonology, Hospital Santa Creu i Sant Pau, Barcelona, Spain; 6 Primary Care Center Francia, Fuenlabrada, Madrid, Spain; 7 Department of Pulmonology, Hospital Santa Creu i Sant Pau, Barcelona, Spain

**Keywords:** Asthma control, GINA guidelines, Asthma control questionnaire, Validation study

## Abstract

**Introduction:**

Asthma Control Questionnaire (ACQ) is a validated tool to measure asthma control. Cut-off points that best discriminate “well-controlled” or “not well-controlled” asthma have been suggested from the analysis of a large randomized clinical trial but they may not be adequate for daily clinical practice.

**Aims:**

To establish cut-off points of the ACQ that best discriminate the level of control according to Global Initiative for Asthma (GINA) 2006 guidelines in patients with asthma managed at Allergology and Pulmonology Departments as well as Primary Care Centers in Spain.

**Patients and methods:**

An epidemiological descriptive study, with prospective data collection. Asthma control following GINA-2006 classification and 7-item ACQ was assessed. The study population was split in two parts: 2/3 for finding the cut-off points (development population) and 1/3 for validating the results (validation population).

**Results:**

A total of 1,363 stable asthmatic patients were included (mean age 38 ± 14 years, 60.3% women; 69.1% non-smokers). Patient classification according to GINA-defined asthma control was: controlled 13.6%, partially controlled 34.2%, and uncontrolled 52.3%. The ACQ cut-off points that better agreed with GINA-defined asthma control categories were calculated using receiver operating curves (ROC). The analysis showed that ACQ < 0.5 was the optimal cut-off point for “controlled asthma” (sensitivity 74.1%, specificity 77.5%) and 1.00 for “uncontrolled asthma” (sensitivity 73%, specificity 88.2%). Kappa index between GINA categories and ACQ was 0.62 (p < 0.001).

**Conclusion:**

The ACQ cut-off points associated with GINA-defined asthma control in a real-life setting were <0.5 for controlled asthma and ≥1 for uncontrolled asthma.

## Introduction

Asthma is a global health problem that affects around 300 million individuals of all ages, ethnic groups, and countries [1]. It is estimated that 250,000 people die prematurely each year as a result of asthma [2]. Proper care of patients with asthma involves the triad of systematic chronic care plans, self-management support, and appropriate medical therapy [3,4].

The Global Initiative for Asthma (GINA), in the 2006 update proposed a new classification of asthma, based on the level of control rather than the previous classification based on the severity of the underlying disease process [5]. Thus, asthma treatment goal, regardless of patient's asthma severity, should lead to achievement of complete disease control. In addition, prevention of future risks should also be intended [6,7].

The main limitation of GINA classification of asthma control is that it is based on an expert consensus, and has not yet been validated in real world practice [8]. Nevertheless and despite the availability of guidelines, a substantial proportion of adults as well as children with asthma are not optimally controlled [9-12].

Standardized and validated questionnaires, such as the Asthma Control Questionnaire (ACQ), help us to assess the level of asthma control [13]. However, the ACQ cut-off points previously defined to discriminate between “well-controlled” and “not well controlled” asthma have been suggested from the analysis of a large randomized clinical trial (Gaining Optimal Asthma Control –GOAL- study), and they may not be adequate for daily clinical practice [14]. In addition, several variables such as rhinitis, exposure to tobacco smoke, obesity and allergen exposure influence as well asthma control [15-17].

The primary aim of the present study was the establishment of the ACQ cut-off points that best discriminate the degree of asthma control according to 2006 GINA criteria in a population of patients with asthma from Allergy and Pulmonology Departments as well as Primary Care Centers. The secondary aim was to validate the results obtained from the development population in another sample population.

## Patients and methods

### Study design

Multicenter, epidemiological descriptive study, with prospective data collection. Patients with physician-diagnosed asthma were consecutively recruited from among those visiting Allergy and Pulmonology Departments as well as Primary Care Centers. Each participating physician selected the first eight patients who met the inclusion criteria during a six month period. Data were collected in a case report form completed by the physician. Patients were required to give written informed consent before inclusion in the study. The study protocol was approved by the institutional review board and the study was conducted in accordance with the principles of the Declaration of Helsinki.

### Patients

Patients > 12 and < 65 years of age diagnosed with asthma and with a stable clinical condition* that attended one of the participant Allergy and Pulmonology Departments or Primary Care Centers during a six month period were eligible to participate in the study. Other inclusion criteria included administration of asthma treatment within the month prior to study inclusion, and absence of any psychological, psychical or language limitation that prevent the correct completion of the case report form.

#### Definition of asthma diagnosis

The medical record must contain description of symptoms consistent with asthma and objective evidence of variable airway obstruction, following the diagnostic criteria of GINA guidelines [5]. Additionally a demonstration of a positive bronchodilator test (increase in FEV_1_ ≥12% and 200 mL) on at least one occasion within the previous year was required.

#### *Definition of stable clinical condition

Patient had not required hospital admissions, ER visits or timely use of oral corticosteroids within the last month.

### Study aims

#### Primary aim

The primary aim of the study was the establishment of cut-off points to discriminate the level of asthma control (as defined in GINA 2006 guidelines) using the ACQ questionnaire, in asthmatic patients attending Allergy and Pulmonology Departments as well as Primary Care Centers.

GINA-defined asthma control: According to clinical characteristics that include daytime symptoms, limitations of activity, nocturnal symptoms/awakening, need for reliever/rescue treatment; lung function (PEF or FEV_1_), asthma patients were classified as controlled, partially controlled or uncontrolled [5]. Thereafter, treatment management was based on the level of asthma control [5].

ACQ: Contains five items scoring symptoms, a question about frequency of β_2_-agonists use and another about pre-bronchodilator FEV_1_ (%) (total of seven questions) [13]. Patients are asked to score how their asthma has been in the previous 7 days and respond to each question on a 7-point scale (0 = no impairment; 6 = maximum impairment). Scores range between 0 (well controlled) and 6 (extremely poorly controlled). A validated Spanish version of the questionnaire was used [18]. In addition, a validated simplified version of the questionnaire (ACQ-5), in which FEV_1_ and β2-agonist use questions are excluded from the seven-item ACQ was also used for analysis [19].

#### Secondary aim

Validation of the primary aim of the study by performing the same analysis in a different sample of the patient population.

### Variables analyzed

Patients completed two self-administered questionnaires (ACQ and Mini Asthma Quality of Life Questionnaire – MiniAQLQ [13,20]) and answered one question regarding his/her own asthma control perception (well controlled, partially controlled or uncontrolled asthma) before entering the physicians’ office. Physicians, who were blinded to the results of the previous tests, completed an electronic case report form which recorded the following patients’ epidemiological and clinical variables: age, gender, height, weight and BMI (kg/m^2^) smoking status; place of residence (rural or urban); contact with animals; prior history of atopic disease, rhinitis, conjunctivitis, atopic dermatitis, urticaria, food allergy; family history of atopic disease; time since asthma diagnosis; maintenance asthma treatment; number of asthma exacerbations within the last year; asthma and rhinitis comorbidity; time since rhinitis diagnosis; severity of rhinitis according to ARIA classification [15]; treatment for rhinitis, and other data related to rhinitis, atopy, etc., that will be published elsewhere.

In the electronic data collection form, the 6 items included in the GINA classification of control [5] were included and analyzed to evaluate the category of control, which was the comparator or gold standard.

Physicians also gave their own perception of patient’s level of asthma control (well controlled, partially controlled or uncontrolled asthma).

### Statistical analysis

#### Sample size

The sample size was calculated for a sensitivity and specificity of at least 75%, with a confidence level of 95% and a sampling error of 5%. This would require a sample size of 180 physicians and 8 consecutive medical patients (total sample: 1,440), with an estimated percentage of follow up loss of 20%. The results were validated in a subpopulation of 402 patients.

#### Statistical analysis

For the description of continuous variables, the mean and standard deviation, the median and the interquartile range in the case of asymmetry and the maximum and minimum values observed were used. For the description of categorical variables, the number and percentage of patients per response category were used. The qualitative variables were compared using the chi-squared test and the quantitative variables using the t-Student test or variance analysis after study of variance homogeneity. Intra questionnaire reliability was analyzed with the frequency for endorsement and the Cronbach’s alpha.

Eligible patients from the entire data base were randomized in a 2:1 manner to create a development and validation datasets, respectively. The development dataset was initially used for evaluating agreement and determining de cut-off points for the ACQ associations with GINA 2006 control classification. The result was tested in the validation dataset.

The inter-method reliability (asthma control questionnaire and GINA 2006 guidelines) was assessed using the kappa or weighted ordinal differing weights for ordinal scores. Receiver operating characteristic (ROC) curves were built to evaluate the discriminative power of the ACQ score over the GINA 2006 clinical guidelines. Both the area under the curve (AUC) and the hypothesis testing were calculated as well as the cut-off points that discriminated between control and no control. Once the cut-off point was selected, the convergent validity was assessed and subsequently compared with ACQ scale through the combination of sensitivity, specificity, and positive and negative likelihood ratios. The 95% confidence intervals were estimated for all the parameters.

For the statistical analysis, the SPSS version 15 for Windows statistical package was used. A level of statistical significance of *p* < 0.05 was be used for all statistical tests performed.

## Results

### Patients’ characteristics

**Figure 1:**
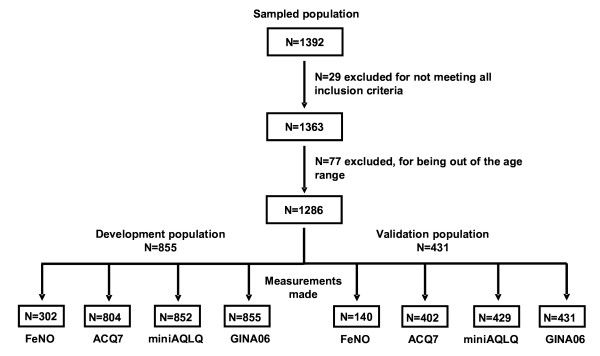
Strobe flow chart.

From January 2009 to July 2009 a total of 1,392 patients from 180 Allergy (34.4%) and Pulmonology (45.2%) Departments from University Hospitals as well as Primary Care Centers (20.4%) were enrolled in the study. Of those, 29 did not fulfill the inclusion criteria and were excluded from the study. Reasons to be excluded were: lack of informed consent (n = 5); absence of asthma diagnosis (n = 1); age out of range (n = 1); has not required asthma medication within the last month (n = 20); unstable clinical condition (n = 1); recent asthma exacerbation (n = 1). Thus, a total of 1,363 patients with asthma were included in the analysis (Figure 1). Baseline patients’ characteristics are depicted in Table 1. Mean age was 37.8 years, approximately 60% of the patients were women; 69% were non-smokers; 67% lived in an urban area and 63% had no contact with animals. The mean duration of asthma and rhinitis was 12.9 and 13.5 years, respectively. In addition, 72.7% of the patients were atopic. Of those, 91.6% presented with rhinitis, 41.9% conjunctivitis, 16.3% atopic dermatitis, 8.8% food allergy and 5.7% urticaria. With regards to rhinitis, most of the patients presented mild intermittent disease (39.4%).

**Table 1:** Baseline patients’ characteristics

**Variables**	**Total population (n = 1,286)**	**Development population (n = 855)**	**Validation population (n = 431)**	***P value***
**Age in years, median, (IQR)**	**38 (27–50)**	**37 (27–48)**	**37 (26–50)**	***0.502***
**Women (%)**	**60.3**	**60.6**	**59.6**	***0.741***
**Smoking habit (%):**				
**Smoker**	**12.8**	**13.0**	**12.4**	***0.322***
**Non-smoker**	**69.1**	**67.9**	**71.7**	
**Ex-smoker**	**18.0**	**19.1**	**15.9**	
**Environment (%):**				
**Urban**	**67.4**	**68.8**	**64.8**	***0.154***
**Rural**	**32.6**	**31.2**	**35.2**	
**Years since asthma diagnosis: Median, (IQR)**	**10 (4.4 - 20)**	**10 (4.5-20)**	**10 (4–19)**	***0.830***
**Rhinitis (% patients)**	**91.7**	**93.2**	**90.1**	***0.780***
**Lung function**				
**FEV**_**1**_**mean% (SD)**	**88.3 (18.8)**	**88.5 (19)**	**88.0 (18.6)**	***0.642***
**FVC mean% (SD)**	**95.5 (19.02)**	**95.4 (19.4)**	**95.7 (18.3)**	***0.813***
**FEV**_**1**_**/FVC mean% (SD)**	**76.7 (13.07)**	**77.2 (13.8)**	**75.9 (11.5)**	***0.08***

### Pulmonary function tests

Spirometry was conducted the day the patient was included in the study. Results of lung function tests are shown in Table 1. At inclusion, 64.5% of patients had a FEV_1_ ≥ 80% predicted. Mean FEV_1_ for controlled patient was 99.2% ± 13.8 and 95.8% ± 14.9 for partially controlled patients. Uncontrolled patient showed a mean FEV_1_ of 81.0% ± 19.0 that was significantly lower compared to controlled or partially controlled patients (p < 0.01).

### Asthma treatment

Maintenance treatment was distributed as follows: Combination of inhaled corticosteroid and long-acting β_2_-agonist (75.7%), antileukotrienes (36.50%), inhaled corticosteroids (12.0%), allergen immunotherapy (13.0%), oral corticosteroids (2.3%), others (8%). Percentage of patients in each step of asthma treatment according to GINA were: Step 1, 9.6%; Step 2, 11.6%; Step 3 + 4, 73%, and Step 5, 5.8%. Patients at step 3 and 4 were merged in one category, as we do not have data about the doses of inhaled corticosteroids.

### Asthma control according to GINA 2006 guidelines and patients’ and physicians’ perceptions

**Figure 2:**
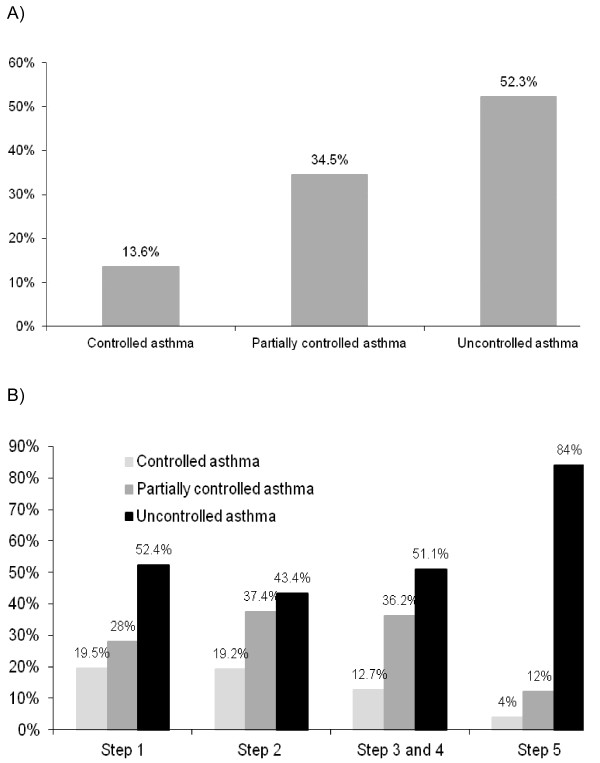
**Asthma control: 2a) According to GINA 2006 guidelines and 2b) In each step of the treatment.2a)** Asthma control according to GINA 2006 (N = 855). **2b)** Asthma control according to treatment step (N = 624).

**Figure 3:**
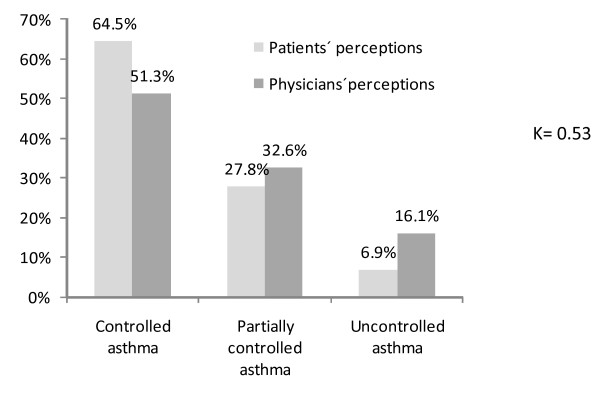
Perception of asthma control according to patients’ and physicians’ perceptions.

More than half of the patients (51.4%) had uncontrolled asthma according to GINA 2006 classification (Figure 2a). Moreover, as shown in Figure 2b, approximately half of the patients in treatment steps 1 through 4 had uncontrolled asthma. Although most of both patients and physicians perceived the disease as controlled or partially controlled, the concordance among both groups was low (κ = 0.53) (Figure 3).

### Establishment of cut-off points required to discriminate the level of asthma control (GINA definition) using the ACQ questionnaire

**Figure 4:**
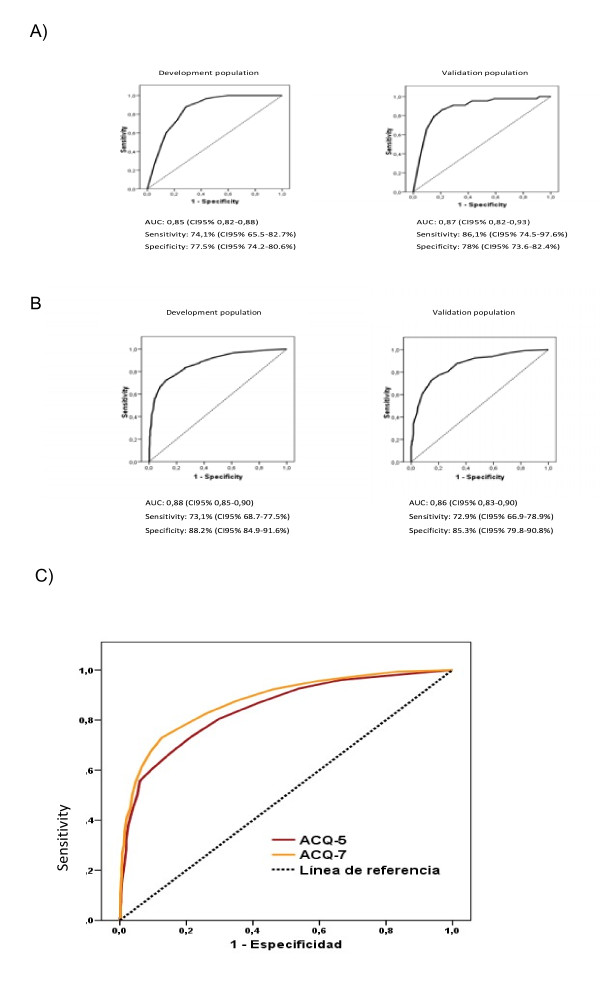
**ACQ cut-off points obtained in the development and validation population.4a)** Uncontrolled vs.controlled. **4b)** Uncontrolled vs. partially controlled. **4c)** ACQ-7 vs ACQ-5.

In the development population (n = 804) the cut-off point of the ACQ questionnaire that better discriminated the controlled asthma patients was 0.5, with a sensitivity of 74.1% (95%CI, 65.5-82.7) and a specificity of 77.5% (95%CI, 74.2-80.6) (Figure 4a). On the other hand, the cut-off point that better discriminated the uncontrolled asthma patients was 1.00, with a sensitivity of 73% (95%CI, 68.7-77.5) and a specificity of 88.2% (95%CI, 84.9-91.6) (Figure 4b).

### Validation study

These results were validated in a subpopulation of 402 patients (Figure 1). The results in the validated population showed a similar value for controlled asthma with a cut-off point of 0.5 with a sensitivity of 86.1% (95%CI, 74.5-97.6).and a specificity of 78% (95%CI, 73.6-82.4) (Figure 4a) and a cut-off point of 1.00 for uncontrolled asthma, with a sensitivity of 72.9% (95%CI, 66.9-78.9) and a specificity of 85.3 (95%CI, 79.8-90.8) (Figure 4b). The AUC in the development population for controlled and uncontrolled asthma were 0.85 (95%CI, 0.82-0.88) and 0.88 (95%CI, 0.85-0.90), respectively p < 0.0001. In the validated population, the AUC for controlled asthma was 0.87(95%CI, 0.82-0.93), and for uncontrolled asthma 0.86 (95%CI 0.83-0.90) (Figure 4 a and b). Similar results were obtained when the simplified questionnaire (ACQ-5) was used (Figure 4c).

### Concordance between GINA 2006 guidelines and ACQ scores

Kappa index between GINA categories and ACQ cut-off points derived from the study was 0.62 (p < 0.001). As a theoretical exercise a low concordance was found between GINA-defined control categories and the former cut-off points of the ACQ questionnaire suggested by Juniper et al. [14] (κappa index = 0.27).

## Discussions

The recent changes in the management of asthma, based on disease control rather that degree of severity, have created the need of new evaluations of the current available tools for measuring asthma control. Among the validated and standardized questionnaires are the ACQ [13], the Asthma Control Test (ACT) [21] and Asthma Therapy Assessment questionnaire (ATAQ) [22]. The ACQ questionnaire was chosen in the present study because has strong evaluative and discriminative properties, is short and easy to complete and can be used with confidence to measure asthma control in the past week, therefore reducing the recall bias.

The results of the present study show a poor correlation between the cut-off points described by Juniper et al. [14] and GINA 2006 control classification. It is worth remarking, however, that in the study of Juniper et al. [14] the definitions of control were based on the GOAL study [23], and these authors grouped well-controlled and total control as controlled, and did not attempt to distinguish between three categories. Juniper et al. adopted the conservative stance that “the crossover point between ‘well controlled’ and ‘not well controlled’ is close to 1.00 on the ACQ. However, to be confident that a patient has well-controlled asthma, the optimal cut-point is 0.75 (negative predictive value = 0.85). To be confident that the patient has inadequately controlled asthma, the optimal cut-point is 1.50 (positive predictive value = 0.88). In clinical practice, however, instead of the value 1, these authors advise that, to make sure that most patients with inadequately controlled asthma are not missed; the optimum cut-point is 0.75 where there is an 85% chance that his/her asthma is well controlled [14].

Based on the calculations using the population of the present study, the cut-off points of the ACQ questionnaire that best agree with the levels of control proposed by GINA 2006 are: < 0.5 for controlled asthma; 0.5 – 0.99 for partially controlled asthma, and ≥ 1 for uncontrolled asthma. Moreover, the same cut-off points were obtained with the simplified version of the ACQ questionnaire (ACQ-5), which is based exclusively in clinical evaluation, avoiding the FEV_1_ measurement as well as β_2_-agonists rescue use, which should facilitate its use in primary care setting. It should be emphasized, however, that ACQ cut-off points must be based on an understanding on how representative the study population is of the population intended to take the test. It is also worth underlying that ACQ, and control assessment in general, is intended for the maintenance of asthma control in the ongoing management of the disease.

Similar discrepancies have been observed in different studies where ACQ or ACT questionnaires have been used as a predictor of GINA-defined asthma control [8,24] . Thus, the study by Alvarez-Gutiérrez et al. [24] found different cut-off points for ACT than previously described (≤ 18 for uncontrolled asthma), suggesting that a more complete assessment would require monitoring operating parameters and FeNO. In the study published by Thomas et al. [8], a multinational cross-sectional survey conducted in Primary Care, Pulmonology and Allergology settings from France, Germany, Italy, Spain and US, and in Primary Care and Pulmonology departments in UK, a total of 2,949 patients filled out the ACT and physician’s case report forms with questions related to GINA classification without spirometry. A score of ≥20 for the ACT score defined well controlled asthma (positive predictive value of 51.3%) while a score of ≤ 19 defined partially controlled and uncontrolled asthma (positive predictive value of 93.9%).

The study by Sastre et al. [25] also recommended the use of different cut-off points to define well-controlled asthma using three versions of ACQ. In addition, O’Byrne et al. [26]., compared asthma control as assessed by the Asthma Control Questionnaire (5-item version; ACQ-5), Global Initiative for Asthma (GINA) or Gaining Optimal Asthma Control (GOAL) study criteria in a retrospective study. The results showed that patients with controlled, partly controlled and uncontrolled asthma according to GINA had mean ACQ-5 scores of 0.43, 0.75 and 1.62, respectively.

However, all these studies were retrospectively designed and not specifically developed for addressing such correlation and, therefore, have important limitations. Moreover, comparing a categorical with a continuous variable is bound to give some difficulty, especially for clinicians.

With regard to measurement of disease control, the results also show that ACQ questionnaire is a more accurate tool compared to the subjective perception of both physicians and patients. It is also noticeable the poor correlation found between physicians’ and patients’ perception of disease control as observed in prior studies [27]. The information efflux fact used in the present study, where patients completed the ACQ and miniAQLQ before seeing the physician, is the best way to collect patients’ opinion avoiding the risk of physician opinion bias.

Asthma control achieved in the present study (conducted between February and July 2009) was suboptimal, with 51.3% of patients being uncontrolled according to GINA, despite the broad use of different treatments, as corroborated in prior studies. In addition, some of the prior studies have shown even worse control in winter season than in spring [9]. The present study did not take into account seasonal variations, as the majority of the patients were included in the study during spring season and hence our results mainly reflect asthma control in the spring.

There is considerable room for improvement regarding management of the disease from the physician perspective through training interventions as shown in the study conducted by Mendez et al. [28]. In addition, patient information with regards to disease control could and should be enhanced by providing more information during the doctor’s visit. In primary care settings several studies show that ACQ seems to be a useful starting point for health care professionals in family practice to improve the level of asthma symptom control in their patient population [29].

When analyzing the asthma treatment prescribed to the patients in the present study, is surprising the high percentages of patients classified as having GINA-defined uncontrolled disease (52.3%), and those receiving combination treatment (78.8% were on step 3 or higher). The results confirm the low degree of asthma control in Spain, which may be due to several factors, including low degree of implementation and compliance with asthma management guidelines, poor adherence to asthma treatments [30], among others. However, it should also be considered that the GINA scale to identify controlled, partially controlled or uncontrolled asthma was developed based on expert opinion and is not validated. Controlled asthma, as defined in GINA, is quite similar to “totally controlled” asthma defined in the GOAL study [23], and this turns out to be a maximal therapeutic goal that is difficult to achieve for many patients. GINA-defined “partially controlled” is a more reasonable therapeutic goal. Interestingly, a retrospective analysis of three clinical trials with combination therapy showed that controlled and partly controlled asthma by GINA criteria are comparable to an ACQ-5 score of <1 [26]. Our study reinforces this finding, with the strength that it was analyzed prospectively in a clinical practice setting.

Although participant physicians were mainly in specialized settings (79.6% from Allergy and Pulmonology settings vs. 20.4% from primary care), the same results regarding asthma control were obtained when the analysis was stratified by this variable (data not shown). This homogeneity in the results is noticeable because the severity of the disease of the patients seen in the three physician groups is different, according to published epidemiological studies [31].

In conclusion, the results of the present study provide new cut-off points for ACQ questionnaire with a better agreement to the asthma control classification proposed by GINA 2006, which can be applied to asthmatic patients in clinical practice. Finally, the short version of the ACQ (ACQ-5), measures very easily the degree of asthma control in primary care as well other specialized settings.

## Competing interest

Dr Juliá de Páramo is an MSD employee. The other authors have declared no conflicts of interest.

## Authors’ contributions

JO and SQ initially proposed the idea and contributed equaly to the design, data analysis and writing of the study. BJ participated in the design of the study, data analysis and writing. CF made the statistical design and analysis. AMF evaluated the initial draft and final protocol. JM evaluated the initial draft and final protocol. VP participated in the design of the study, final protocol and data analysis. All authors read and approved the final manuscript.
